# A machine learning approach to predict postoperative sleep disturbance after total knee arthroplasty: a comparative study of multiple algorithms

**DOI:** 10.3389/fmed.2025.1699842

**Published:** 2025-11-05

**Authors:** Yi-xiang Zhang, Sen He, Tao Yang, Hao-liang Li, Chun-lei Wu, Lei Wang, Xiao-quan Wang, Jun Liu

**Affiliations:** ^1^College of Orthopedics, Tianjin Medical University, Tianjin, China; ^2^Department of Joints, Tianjin Hospital of Tianjin University (Tianjin Hospital), Tianjin, China; ^3^Department of Joints, Beichen Hospital, Tianjin, China; ^4^The United Logistics Support Force of 983 Hospital, Tianjin, China

**Keywords:** postoperative sleep disturbance, total knee arthroplasty, machine learning, predictive model, SHAP analysis

## Abstract

**Background:**

Postoperative sleep disturbance (PSD) is a common complication following total knee arthroplasty (TKA), which negatively impacts patient recovery. Despite the critical need for early detection and management, there is limited research on predictive models for early PSD, particularly those integrating machine learning (ML) techniques.

**Objective:**

This study aimed to develop a predictive model for early PSD following TKA using ML algorithms, identify key predictive factors, and provide an interpretable model to guide clinical decision-making.

**Methods:**

The study included 505 patients who underwent TKA. Clinical data were collected at three stages: preoperatively, intraoperatively, and postoperatively. Ten MLa models, including logistic regression, support vector machine (SVM), and XGBoost, were trained and evaluated using a test set. Performance metrics, including accuracy, sensitivity, specificity, and area under the curve (AUC), were used to evaluate the efficacy of the models. Key features influencing PSD were identified through SHapley Additive Explanations (SHAP) analysis to enhance model interpretability.

**Results:**

Gradient Boosting Machine (GBM) demonstrated the highest AUC (0.906), accuracy (0.834), and sensitivity (0.879), establishing it as the optimal model for predicting PSD. Key predictors identified included age, smoking, living alone, living in the city, VAS 1 month postoperative, and anxiety 1 month postoperative. SHAP analysis revealed that postoperative VAS and age were the most influential factors in predicting PSD, with their impact varying based on individual patient data.

**Conclusion:**

The study developed a robust and interpretable ML model for the early prediction of PSD following TKA. This model can aid in preoperative risk stratification, facilitating personalized management strategies to improve postoperative outcomes. Further validation in larger cohorts and diverse settings is necessary to enhance its broader clinical applicability.

## Introduction

Knee osteoarthritis (OA) is a common musculoskeletal disorder that causes significant pain and dysfunction (1). TKA is an effective treatment for end-stage knee OA, and its use is increasing due to the aging population and advancements in surgical techniques (2–4). TKA is highly effective in relieving pain, improving joint function, and enhancing quality of life; however, up to 20% of patients remain dissatisfied after the procedure (5–8). The etiology of patient dissatisfaction after TKA is multifactorial. Sleep disorders, particularly postoperative sleep disturbance (PSD), are increasingly recognized as common and detrimental to recovery after TKA (9). These disorders can negatively affect postoperative pain management, mental health, and overall recovery (10, 11).

Postoperative sleep disturbance is a common but often underrecognized complication following surgical procedures. Studies suggest that the incidence of perioperative PSD in patients undergoing TKA may exceed 50% (12). PSD is characterized by difficulty falling asleep, fragmented sleep, frequent nocturnal awakenings, and poor sleep quality (9). PSD not only causes patient dissatisfaction but also has significant negative consequences on postoperative recovery (13). PSD is strongly associated with worsened pain perception, complicating postoperative pain management and delaying recovery (14–16). Additionally, PSD is linked to increased levels of anxiety and depression, further hindering rehabilitation and prolonging recovery (17). Furthermore, poor sleep quality impairs immune function, delays wound healing, and increases the risk of postoperative complications, resulting in longer hospital stays and higher healthcare costs (18–20).

Current research on PSD predictors primarily relies on traditional statistical approaches, particularly logistic regression (21). While valuable, these methods depend on pre-specified linear assumptions and struggle to capture the complex, non-linear interactions among numerous clinical, psychological, and social factors influencing postoperative sleep. This limitation necessitates analytical approaches that can automatically learn these complex patterns from data. ML has shown remarkable potential in this regard, revolutionizing predictive modeling across various medical specialties with its robust data processing capabilities and superior predictive performance (22, 23). Despite these advancements, however, the application of ML to PSD prediction following TKA remains underdeveloped, with a notable scarcity of dedicated models in the current literature.

This study aims to address this critical research gap by developing and validating a comprehensive ML-based predictive model for PSD following TKA. Our investigation includes preoperative, intraoperative, and early postoperative clinical data to identify key predictive factors. A fundamental innovation of our approach is the integration of novel socio-environmental predictors, such as “living alone” and “urban residence,” which have been largely overlooked in previous research despite their potentially significant impact on sleep quality during recovery. Through interpretability analysis, we identify key predictive factors for PSD occurrence. Our findings are expected to enable early identification of at-risk patients, support preoperative risk stratification, improve perioperative management, and ultimately facilitate personalized rehabilitation strategies after TKA.

## Materials and methods

### Study design and patient selection

This study was approved by the Institutional Review Board (IRB) of Tianjin Hospital (IRB 2024 Medical Ethics Review 102). All procedures involving human participants were conducted in accordance with the ethical standards established by the IRB and the Declaration of Helsinki. All participants signed informed consent forms, explicitly stating that their clinical data would be used for research and model development. Additionally, all data were de-identified during use to ensure patient privacy and security.

This study included patients who underwent TKA at the Department of Joint Surgery, Tianjin Hospital, between May 2024 and March 2025 for retrospective analysis. The Pittsburgh Sleep Quality Index (PSQI), a widely used self-assessment tool for sleep evaluation, reflects sleep status and quality over the past month. A total PSQI score above 5 indicates poor sleep quality (24). A previous study reported that the incidence of sleep disturbance at 4 weeks postoperatively was 31% (25). The follow-up period in this study was 1 month postoperatively, with the presence of PSD defined by a PSQI score greater than 5.

**FIGURE 1:**
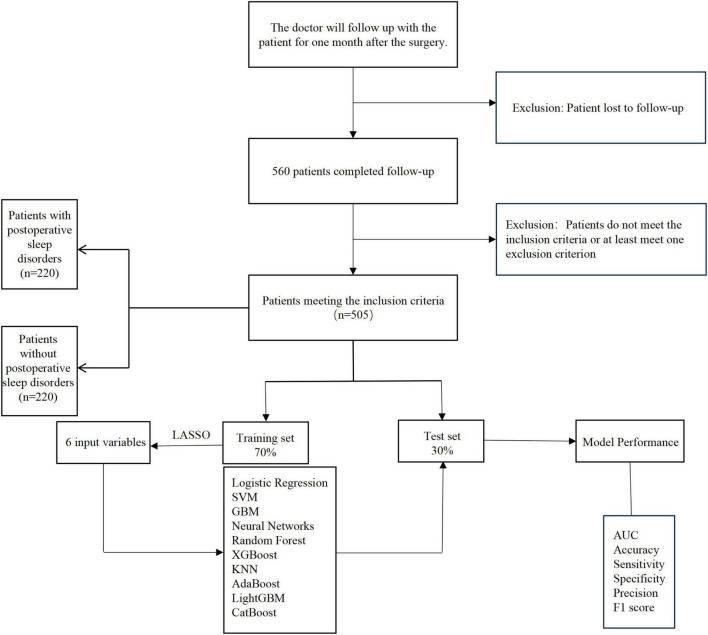
Patient enrollment flowchart.

Importantly, while the PSQI > 5 was used to define the presence of sleep disturbance, this threshold was not used as an inclusion criterion for the study. Instead, all patients who underwent TKA between May 2024 and March 2025 were included in the study regardless of their PSQI scores. Following the application of the exclusion criteria, 505 patients were included in the analysis, with 220 patients diagnosed with PSD (PSQI > 5) and 285 patients not diagnosed with PSD (PSQI ≤ 5) (Figure 1).

The inclusion criteria were: primary osteoarthritis in patients aged 50–80 years undergoing unilateral TKA. The exclusion criteria included: (1) patients with preoperative sleep disturbances (PSQI > 5), (2) severe cognitive or psychiatric disorders, (3) regular use of sleep aids during the perioperative period, (4) prior treatment with other systemic psychological interventions, and (5) >20% missing clinical data.

### Data collection and data preprocessing

The majority of the data were derived from the electronic patient record (ePR) system at Tianjin Hospital and its associated Clinical Data Analysis and Reporting System (CDARS), with the remaining data obtained from postoperative follow-up. As this was a retrospective study, PSQI scores were collected as part of routine clinical care at preoperative visits and postoperative follow-ups, not prospectively assessed specifically for research purposes. Data with more than 20% missing values were excluded from the analysis (26). A total of 38 variables were analyzed, including demographic data (e.g., age, gender, smoking, alcohol consumption, medical history), laboratory results (e.g., WBC, HB, CR, TP), and 1-month postoperative follow-up data [e.g., visual analogue scale (VAS), WOMAC, anxiety levels]. These variables were selected based on clinical plausibility to form a comprehensive feature set for data-driven prediction modeling of PSD.

Patient-reported outcomes and functional measures, including the VAS for pain (27), the Self-Rating Anxiety Scale (SAS) (28), the Self-Rating Depression Scale (SDS) (29), the WOMAC score (30), and knee range of motion, were assessed and documented by experienced clinicians at four postoperative time points: days 7, 14, 21, and 28 during routine follow-up visits. Assessments were conducted using standardized, validated tools. For analysis, the arithmetic mean of the four measurements was computed for each variable to obtain a representative “1-month postoperative” value. This approach was adopted to improve the reliability of the measurement by reducing the influence of daily fluctuations, thereby offering a more stable estimate of the patient’s typical state during the recovery period. An SAS score >50 indicated mild anxiety, and an SDS score >53 indicated mild depression (28, 29). These instruments were widely recognized and validated in clinical practice. This approach ensured data accuracy and reliability, with evaluations conducted by trained healthcare professionals.

The subsequent data cleaning and preprocessing steps involved standardization and conversion of text descriptions into numerical values to ensure dataset quality and accuracy. Continuous variables were retained in their original form. Binary variables, such as gender, were coded (female = 0, male = 1). PSD patients were classified as “cases,” while non-PSD patients were classified as “controls,” with respective coding of 1 and 0. Missing data for continuous variables were imputed using the expectation-maximization method. Missing values for binary variables were imputed using the mode (Supplementary Table 1). Only variables with missing data less than 20% were imputed, while large amounts of missing data were excluded during the patient selection phase (26). This approach ensured the model was developed with a complete, reliable dataset, without artificially inflating the sample size. The characteristics of the data were summarized in Table 1.

**TABLE 1:** Characteristics of the cohort.

Variables	Non-PSD	PSD	*P*-values
**Demographics**			
Age (years)	71.2 ± 8.0	72.4 ± 4.9	**0.023**
Sex, *n* (%)			0.821
Female	196	151	
Male	88	70	
Smoking, *n* (%)	88 (30.8%)	79 (35.9%)	**0.037**
Drinking, *n* (%)	65 (22.8%)	51 (23.2%)	0.921
Diabetes, *n* (%)	116 (40.7%)	94 (42.7%)	0.430
Hyperlipidemia, *n* (%)	109 (38.2%)	84 (38.1%)	0.988
Hypertension, *n* (%)	110 (38.6%)	87 (39.5%)	0.828
Complication, *n* (%)	96 (33.7%)	70 (31.8%)	0.658
Live alone	28(9.8%)	53(24%)	**0.013**
Live in the city	126(44.2%)	118(53.6%)	**0.022**
**Laboratory findings**			
WBC (10^9^/L)	5.95 ± 1.16	6.10 ± 0.29	0.055
HB (g/L)	132.89 ± 11.99	133.11 ± 10.47	0.780
Cr (mg/dL)	79.65 ± 15.72	80.20 ± 18.16	0.622
TP (g/L)	69.91 ± 5.60	67.30 ± 3.00	0.347
ALB (g/L)	36.63 ± 3.11	40.10 ± 3.24	0.940
UA (μmol/L)	284.07 ± 80.72	285.60 ± 61.09	0.648
D_dimer (mg/L)	0.27 ± 0.10	0.28 ± 0.11	0.302
CRP (mg/L)	5.31 ± 2.90	5.35 ± 0.43	0.827
HDL (mmol/L)	1.33 ± 0.17	1.78 ± 0.22	0.651
LDL (mmol/L)	2.82 ± 0.56	2.77 ± 0.15	0.167
TC (mmol/L)	4.39 ± 0.38	4.40 ± 0.50	0.656
ALT (U/L)	32.55 ± 12.10	32.70 ± 11.66	0.883
GLB (g/L)	24.97 ± 1.91	24.93 ± 2.10	0.821
TB (mmol/L)	7.50 ± 2.13	7.53 ± 2.05	0.758
APTT (s)	30.08 ± 2.33	29.91 ± 2.49	0.434
PT (s)	12.45 ± 0.57	11.28 ± 0.62	0.974
**Clinical data**			
Preoperative PSQI	4.44 ± 1.25	3.69 ± 1.31	**0.035**
Preoperative anxiety	42.86 ± 7.94	42.18 ± 6.12	0.987
Preoperative depression	48.10 ± 10.77	49.25 ± 5.64	0.149
Preoperative VAS	7.07 ± 0.85	6.91 ± 1.17	0.076
Preoperative Womac	173.79 ± 14.33	173.98 ± 8.59	0.872
VAS 1 month postoperative	2.30 ± 0.54	2.46 ± 1.10	**0.034**
Womac 1 month postoperative	46.03 ± 4.03	45.89 ± 2.92	0.558
Anxiety 1 month postoperative	37.27 ± 4.78	38.32 ± 5.86	**0.027**
Depression 1 month postoperative	15.80 ± 2.99	15.94 ± 3.09	0.596
One month postoperative knee range of motion	101.05 ± 8.83	105.37 ± 6.18	**0.041**

Bold values indicate statistically significant differences with a *p*-value <0.05. Group comparisons were made using Student’s *t*-test for normally distributed continuous variables, Mann-Whitney U test for non-normally distributed continuous variables, and Chi-square test for categorical variables, as detailed in the Section “Statistical analyses and model development”.

### Statistical analyses and model development

This study began with data preparation and anonymization, followed by preliminary cleaning, which involved removing duplicates and imputing missing values. To develop the predictive model, all preprocessed variables were incorporated directly into a Least Absolute Shrinkage and Selection Operator (LASSO) model for training.

Least Absolute Shrinkage and Selection Operator was chosen because it simultaneously performed feature selection and model fitting. The model applied L1 regularization, shrinking the coefficients of less important features to zero, thereby automatically identifying the most influential variables and preventing overfitting (31). This approach avoided biases associated with pre-selection filtering methods and allowed the model to capture complex multivariate relationships.

Candidate variables were initially screened using univariate analysis (*p* < 0.05). The optimal regularization parameter (λ) was then determined through 10-fold cross-validation, applying the “one standard error” rule (lambda.1se). This criterion selected the most parsimonious model, where the performance was within one standard error of the minimum binomial deviance, thereby favoring model simplicity and robustness.

The dataset was randomly divided into a training set (70%) and a test set (30%) based on common practices in predictive modeling. While this approach was widely used, alternative techniques such as bootstrapping or cross-validation could be considered in future studies to further validate the robustness of the model. The training set was used for model development and hyperparameter optimization, whereas the independent test set was reserved solely for the final evaluation of model performance. For model development, we employed ten ML algorithms: Logistic Regression, support vector machine (SVM), Gradient Boosting Machine (GBM), Neural Networks, Random Forest, eXtreme Gradient Boosting (XGBoost), K-Nearest Neighbors (KNN), AdaBoost, Light Gradient Boosting Machine (LightGBM), and Categorical Boosting (CatBoost). These ten models were selected to ensure comprehensive coverage of major, high-performing machine learning families, including linear models, support vector machines, tree-based ensembles, and boosting algorithms (32). This approach guaranteed a robust and representative comparison of state-of-the-art techniques applicable to structured clinical data. While deep learning approaches were considered, they were not adopted due to the moderately-sized dataset, which was suboptimal for training complex deep networks, and our emphasis on model interpretability for potential clinical use.

Each model was trained using 10-fold cross-validation to assess performance, and hyperparameters were optimized using Bayesian optimization to improve predictive accuracy. The performance of all models was evaluated at each iteration using multiple metrics: AUC, accuracy, sensitivity, specificity, and F1 scores. AUC was prioritized as the primary evaluation metric because it provides a more comprehensive measure of model discrimination, especially in imbalanced datasets (33). AUC represents the area under the curve plotting the true positive rate against the false positive rate, reflecting the model’s predictive ability. The AUC ranges from 0 to 1. Models with an AUC greater than 0.7 are considered to exhibit good performance and clinical significance, with an AUC of 1 representing perfect performance (34). For the remaining metrics, values range from 0 to 1, with higher scores indicating better performance. Given the imbalanced nature of the classification task, AUC and balanced accuracy were emphasized during performance evaluation. The average score across iterations determined each model’s final performance. Among the ten models, the one with the highest AUC was selected as the final model.

To enhance the transparency and interpretability of the final predictive model, both global and local interpretations were incorporated. The global interpretation was presented using the SHapley Additive Explanations (SHAP) summary plot, while local interpretations were visualized with SHAP waterfall plots for individual PSD cases following TKA (35, 36). According to the SHAP legend, the larger the absolute value of a SHAP value in the waterfall plot, the greater its impact on the prediction. Furthermore, differences in performance for the same feature across individuals, as shown in the single-sample waterfall plots, may have arisen from individual variability, highlighting the model’s ability to capture subject-specific differences. We then compared the comprehensive performance metrics of the GBM model across key patient subgroups, with particular focus on socio-environmental predictors and gender distribution. Through these subgroup analyses, we aimed to specifically assess potential model bias and better understand the model’s applicability across different patient demographics, thereby providing evidence for its fairness and generalizability. Since different subgroups may have experienced varying degrees of class imbalance which can significantly impact model performance (37), we evaluated multiple metrics including Accuracy, Sensitivity, Specificity, Precision, F1-score, and the AUC to thoroughly assess the model’s performance in these specific populations. The comprehensive analysis provided valuable insights into model fairness and offered targeted data support for personalized treatment strategies.

This study described the characteristics of various datasets and conducted a series of statistical tests. For continuous data, means and standard deviations were used for normally distributed variables, while medians and interquartile ranges were applied to non-normally distributed variables. Categorical data were summarized using frequencies and proportions. Group comparisons were made using the Student’s *t*-test for normally distributed continuous variables, the Mann-Whitney U test for non-normally distributed continuous variables, and the Chi-square test for categorical variables. A two-tailed *p*-value of < 0.05 was considered statistically significant. All statistical analyses and model construction were performed using IBM SPSS Statistics (version 26.0) and R (version 4.4.2).

## Results

### Cohort characteristics

This study included 505 patients, of whom 220 were diagnosed with PSD and 285 were classified as normal. The prevalence of PSD in our cohort was 43.6%. This finding aligns with the established literature, highlighting the substantial burden of this complication in the postoperative period (12). Among the total patient population, 347 (68.7%) were female, and 158 (31.3%) were male. The mean age of the patients was 71.7 ± 6.9 years, with a mean BMI of 22.4 ± 4.4. A total of 167 patients had a history of smoking, and 116 patients had a history of alcohol consumption. Among comorbidities, diabetes mellitus was the most common, affecting 210 patients (41.6%), followed by hypertension in 197 patients (39.0%) and hyperlipidemia in 193 patients (38.2%). Regarding patient residence, 81 patients (16.0%) lived alone, and 244 patients (48.3%) lived in the city. The baseline demographics, along with the results of univariate and multivariate analyses, are presented in Table 1.

### Predictors screened by LASSO regression

**FIGURE 2:**
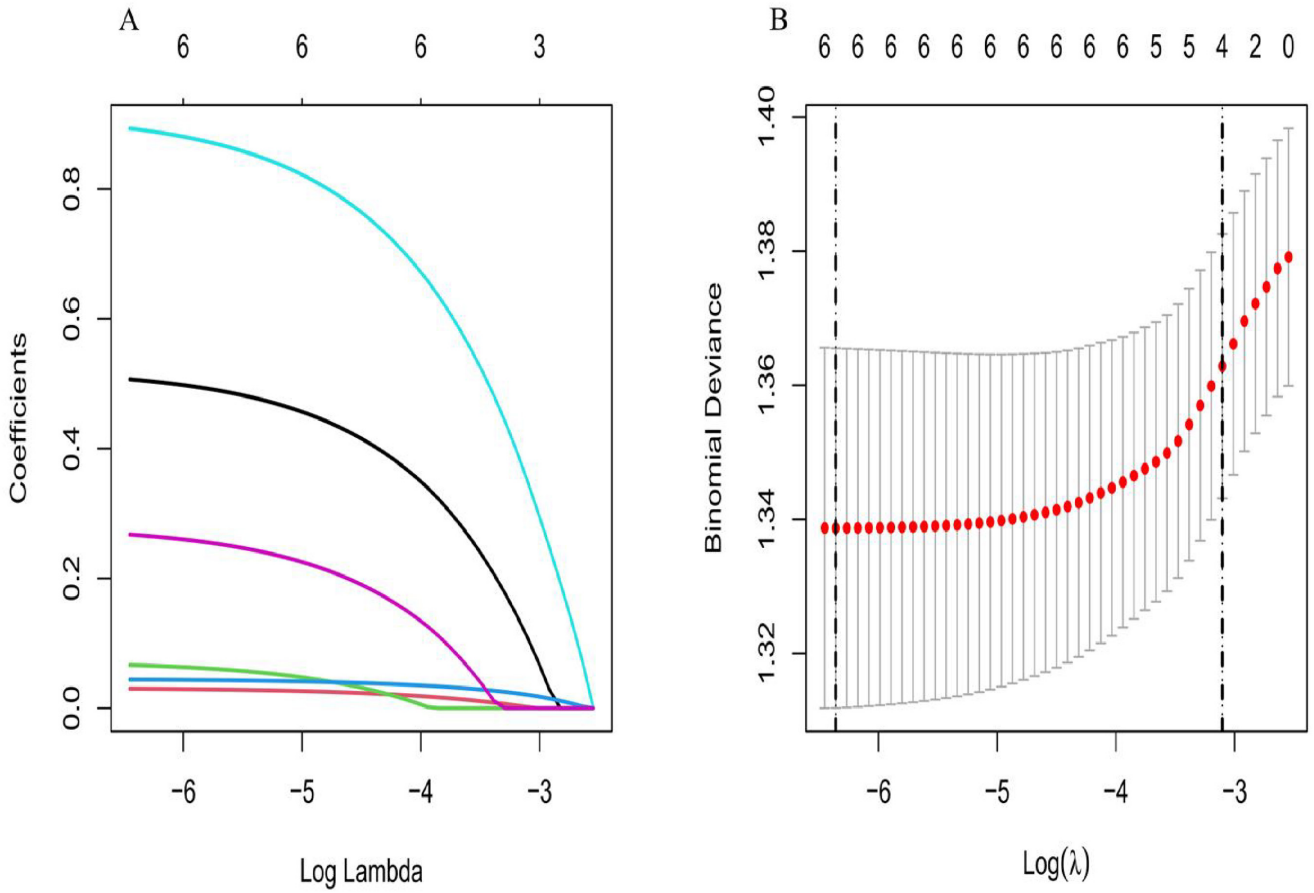
**(A)** Least Absolute Shrinkage and Selection Operator (LASSO) coefficient path plot: This plot shows how the coefficients of different features change as the lambda value increases in a LASSO regression model. As lambda increases, the coefficients of less important features are progressively compressed toward zero. Features that reach zero early contribute less to the model, while those that remain non-zero for a longer period are more influential, indicating their greater relevance in the prediction task. The plot helps in visualizing which features are selected and retained in the model as regularization strength increases. **(B)** Cross-validation curve for LASSO regression: The plot illustrates the binomial deviance (model error) as a function of log (lambda) in a LASSO regression model. The solid curve represents the mean binomial deviance, and the shaded area between the dashed lines indicates the range of one standard deviation above and below the mean. The optimal value of log (lambda) is determined where the error is minimized, corresponding to the lowest deviance, as indicated by the vertical dashed lines. This curve aids in selecting the best regularization parameter for minimizing model error.

Using PSD as the dependent variable, LASSO regression with 10-fold cross-validation identified six key predictors from the initial candidate variables: smoking, age, VAS 1 month postoperative, anxiety 1 month postoperative, living alone, and living in the city (Figures 2A, B). These findings highlight the key risk factors associated with the development of PSD in post-TKA patients, which can assist in clinical decision-making and guide targeted interventions.

### Model performance

**FIGURE 3:**
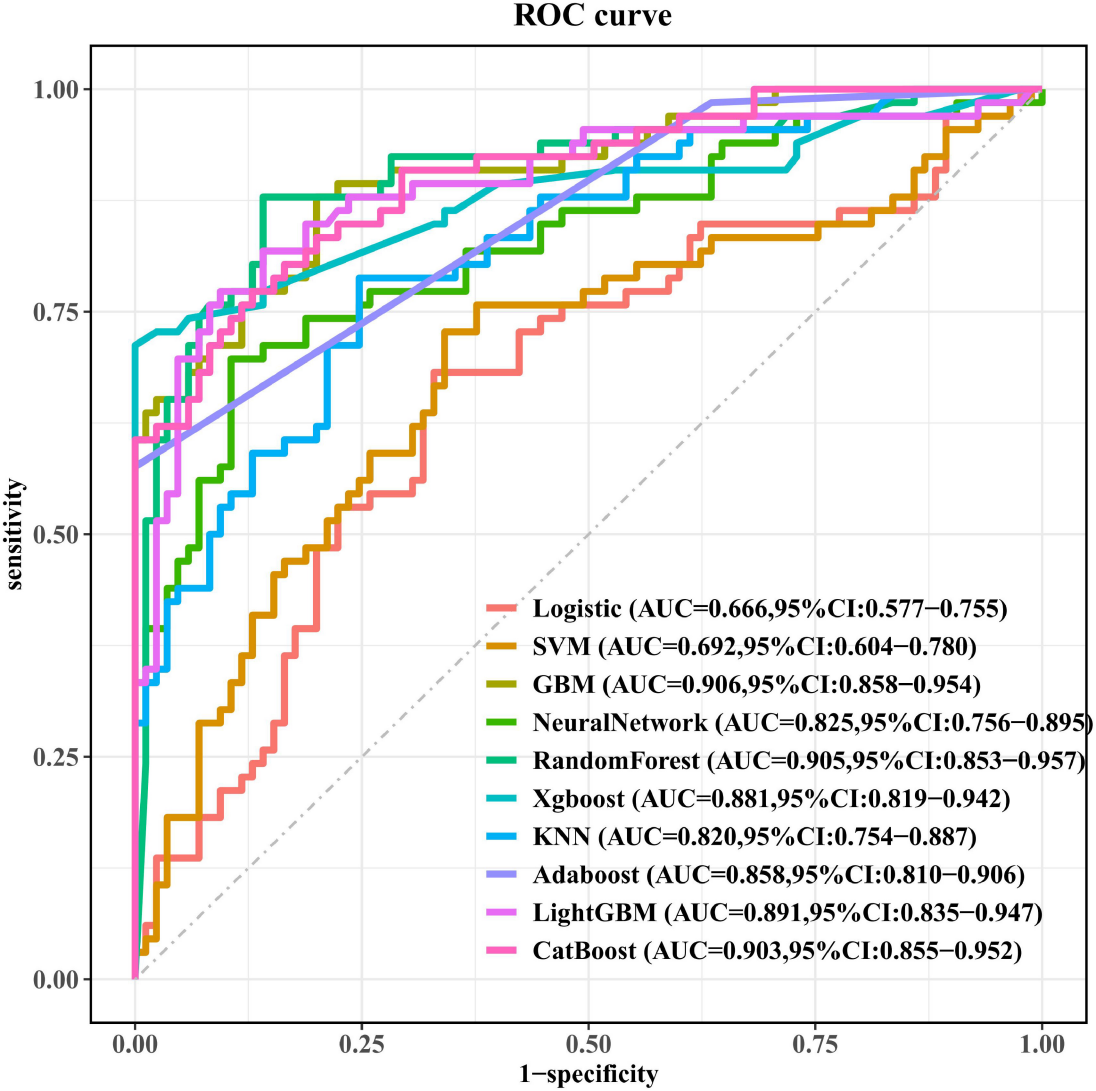
Performance of ten machine learning models on a test set.

The performance of ten ML models was evaluated on the test set, with AUC values ranging from 0.666 to 0.906. Among these models, the Logistic model demonstrated the lowest AUC, while the GBM model achieved the highest AUC, indicating superior discriminative ability. In terms of accuracy, the Logistic model had the lowest value at 0.675, while the XGBoost model achieved the highest accuracy at 0.874. For sensitivity, the AdaBoost model scored the lowest at 0.576. The GBM and Random Forest models achieved the highest sensitivity score of 0.879. For specificity, the LightGBM model performed best, achieving a specificity of 0.906. For precision, the LightGBM model achieved the highest score of 0.864. For the F1 score, the Logistic model scored the lowest at 0.647, while the Random Forest model achieved the highest score at 0.853. Overall, the GBM model demonstrated the best discriminative ability among all ten models and performed consistently and reliably during 10-fold cross-validation. Therefore, the GBM model was selected as the final prediction model (Figure 3 and Table 2).

**TABLE 2:** Performance metrics of different machine learning models for predicting PSD.

Model	Accuracy	Sensitivity	Specificity	Precision	F1
Logistic	0.675	0.682	0.671	0.616	0.647
SVM	0.689	0.727	0.659	0.623	0.671
GBM	0.834	0.879	0.8	0.773	0.823
Neural Network	0.808	0.697	0.894	0.836	0.76
Random Forest	0.868	0.879	0.859	0.829	0.853
Xgboost	0.874	0.712	0.871	0.652	0.832
KNN	0.768	0.788	0.753	0.712	0.748
Adaboost	0.815	0.576	0.775	0.717	0.731
LightGBM	0.848	0.773	0.906	0.864	0.816
CatBoost	0.828	0.773	0.871	0.823	0.797

### Feature importance

**FIGURE 4:**
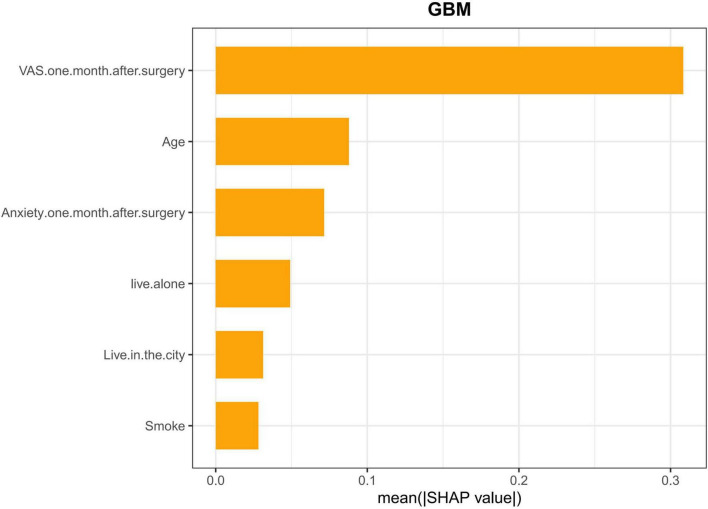
This plot displays a SHAP summary bar chart, ranking each predictor’s average importance in the model’s predictions in descending order. SHAP values represent the contribution of each feature to the model’s prediction. Larger SHAP values indicate a higher impact of the feature on the prediction, while smaller values suggest a lesser influence. From the plot, it is evident that postoperative VAS score 1 month after surgery has the largest impact on the model’s predictions, followed by Age, postoperative anxiety 1 month after surgery, and other features. This plot provides a visual understanding of the relative importance of different features in PSD, helping to identify key factors driving the model’s output.

**FIGURE 5:**
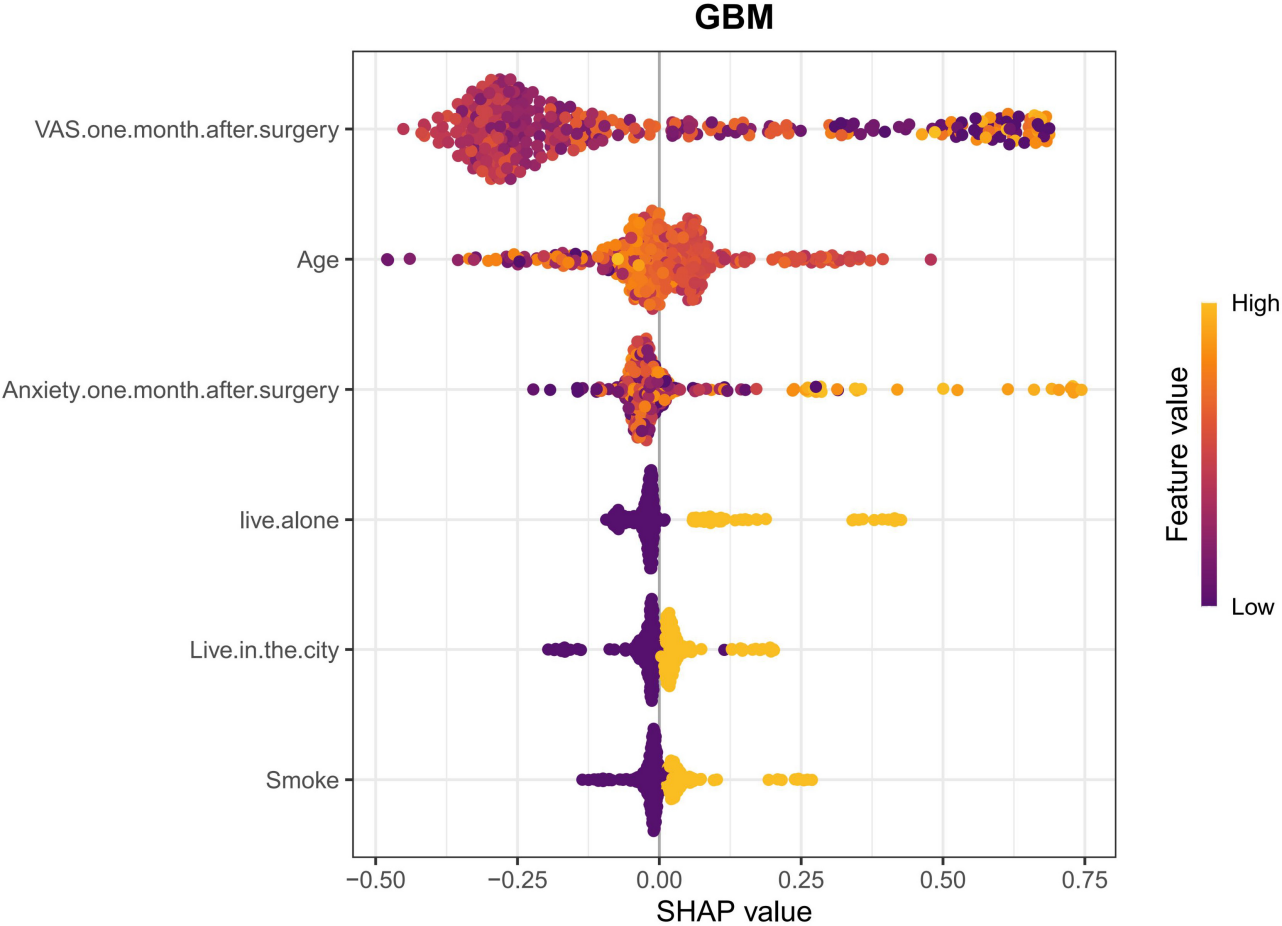
This SHAP summary plot visualizes the influence of key features on the GBM model’s prediction of PSD. Positive SHAP values indicate an increase in the predicted risk for PSD, whereas negative SHAP values suggest a decrease in risk. For continuous features (e.g., VAS 1 month after surgery, Age, Anxiety 1 month after surgery), feature values are color-coded from yellow (low) to purple (high). Generally, higher feature values correspond to a stronger influence on the model’s prediction, with higher VAS scores and Age increasing the predicted risk for PSD. For categorical features (e.g., live alone, Live in the city, smoke), the presence of the feature is represented by yellow (high), and the absence by purple (low), indicating their influence on the predicted outcome. Features with higher SHAP values have a more substantial impact on the model’s output, highlighting their importance in predicting PSD.

SHapley Additive Explanations summary plots offered a global interpretation of model decisions, visualizing the importance of each feature (Figures 4, 5). This analysis confirmed the importance of the six LASSO-selected predictors and further quantified their effects. The model identified VAS 1 month postoperative and age as the most influential factors, followed by anxiety 1 month postoperative, living alone, urban residence, and smoking. Overall, all identified predictors were risk factors for PSD post-TKA.

**FIGURE 6:**
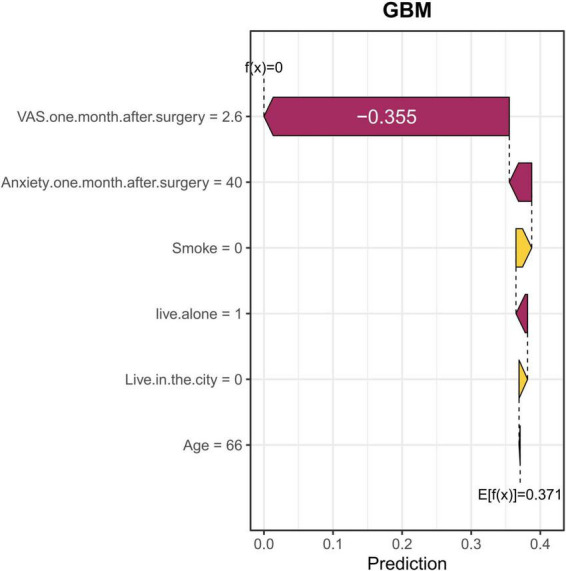
A local SHAP waterfall plot for the 28th TKA patient. This plot illustrates the contribution of each feature to the final prediction for the postoperative sleep disorder model. The length of each bar represents the impact of each feature on the prediction, with red bars indicating a decrease in predicted probability and yellow bars indicating an increase. For this specific patient, the VAS 1 month postoperative value has the greatest negative impact, followed by anxiety 1 month postoperative. Living alone, non-smoking, and not living in cities are important protective factors, all of which contribute to the model’s output. This example demonstrates how the model’s prediction is shaped by different factors, emphasizing the importance of VAS, anxiety, and living alone in influencing the patient’s risk prediction.

**FIGURE 7:**
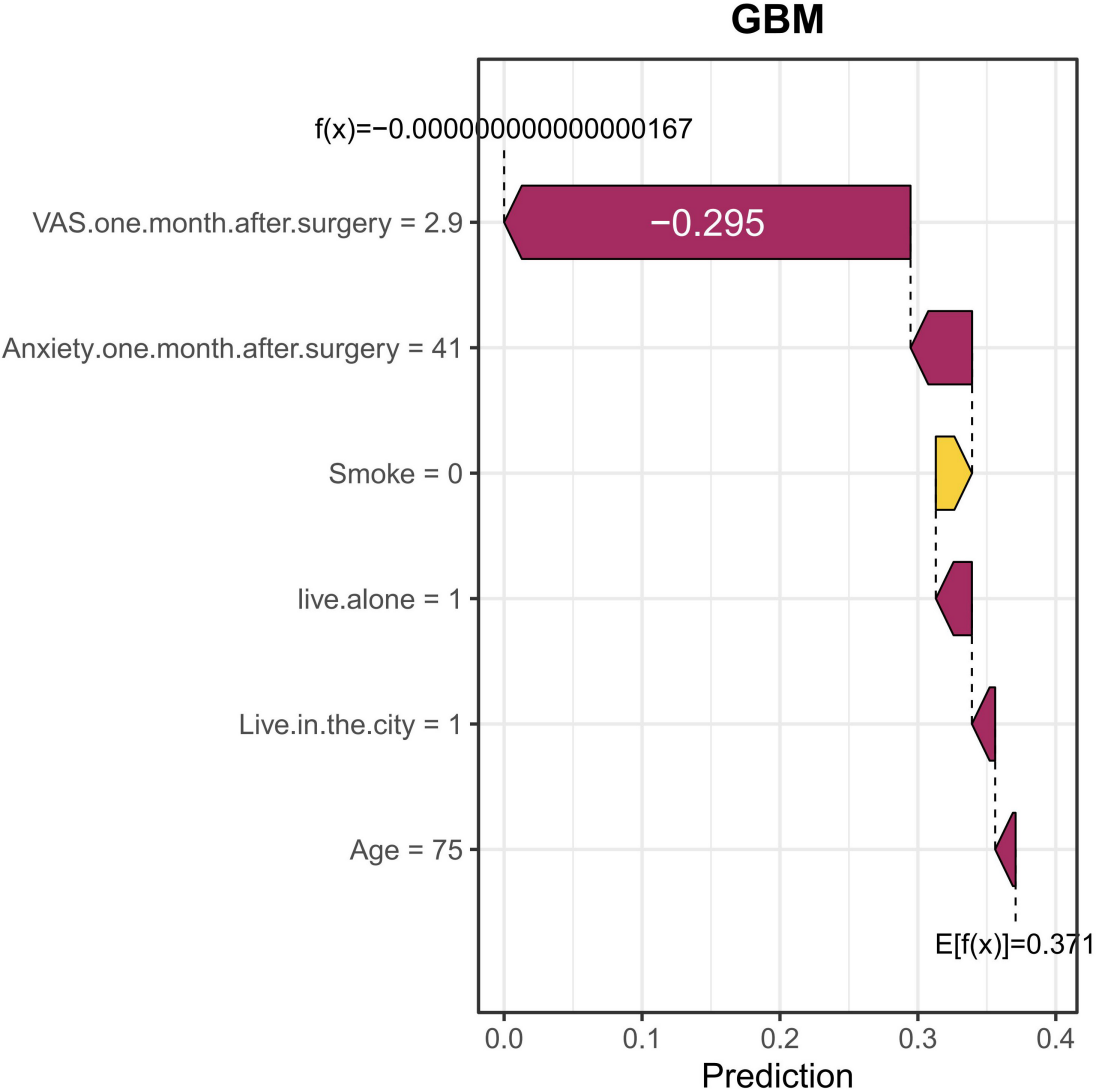
A local SHAP waterfall plot for the 35th TKA patient’s prediction. This plot illustrates the contribution of each feature to the final prediction in the postoperative sleep disorder model. The length of each bar represents the impact of each feature on the prediction, with red bars indicating a decrease in predicted probability and yellow bars indicating an increase. For this specific patient, the VAS 1 month postoperative value has the greatest negative impact, followed by anxiety 1 month postoperative, living alone, living in the city, and age. Non-smoking is an important protective factor, all of which contribute to the model’s output. This example highlights how VAS, anxiety, and smoking influence the model’s prediction of the patient’s risk for postoperative sleep disorders.

We provided two localized SHAP waterfall plots for individual patients to illustrate patient-level interpretations of the final model predictions (Figures 6, 7). Figure 6 shows the 28th TKA patient in our cohort. In this case, VAS 1 month postoperative (2.6) was the most significant risk factor, followed by anxiety 1 month postoperative (38) and living alone. Not smoking and not living in the city were the most important protective factors. Figure 7 shows the 35th TKA patient in our cohort. In this case, VAS 1 month postoperative (2.9) was the most significant risk factor, followed by anxiety 1 month postoperative (39), living alone, and living in the city. Not smoking was the most important protective factor.

### Subgroup analysis

We conducted a detailed subgroup analysis of the final GBM model to evaluate its fairness and generalizability, focusing on social environment and gender factors (Table 3). The model demonstrated strong and consistent predictive performance across most subgroups, with AUC values consistently above 0.88 in gender-based (male/female) and urban residence subgroups. However, performance showed variability in the “living alone” subgroup (*N* = 81). This fluctuation is likely due to the small sample size in this subgroup, which limited the model’s ability to identify stable patterns, combined with a disproportionately high percentage of PSD patients (65.4%), which exacerbated the impact of class imbalance on model stability. These findings suggest that while the model performs reliably overall, caution is warranted when applying it to patients living alone. Future validation with larger sample sizes is necessary to confirm these results.

**TABLE 3:** Performance of the final GBM model across different patient subgroups.

Subgroup	AUC	Accuracy	Sensitivity	Specificity	Precision	F1
**Live in the city**						
Yes	0.910	0.840	0.885	0.805	0.780	0.829
No	0.901	0.828	0.873	0.802	0.766	0.816
**Live alone**						
Yes	0.870	0.790	0.820	0.770	0.740	0.778
No	0.912	0.842	0.885	0.810	0.782	0.883
**Gender**						
Female	0.902	0.831	0.875	0.798	0.770	0.819
Male	0.911	0.839	0.884	0.805	0.781	0.829

## Discussion

This study aims to develop an ML-based model for predicting PSD in patients following TKA. A key innovation of our study is the integration of ten different machine learning models, which offer a multidimensional and comprehensive analytical framework to predict and identify the main risk factors for postoperative sleep disorders. Using machine learning models, we identify two socio-environmental factors—living alone and living in the city—as predictors for the first time, factors that have not received adequate attention in the existing literature. In addition to these two newly identified factors, our study further confirms the importance of clinical factors, such as VAS scores, anxiety symptoms, and age, in predicting PSD.

### Discovery of innovative socio-environmental factors

This study is the first to highlight the significant role of two factors—living alone and living in the city—in predicting PSD. Patients living alone lack care and assistance from family members after surgery, presenting additional challenges during their recovery. The absence of family support, particularly during the postoperative recovery period, often makes it difficult for these patients to manage pain, perform daily activities, and access necessary psychological support (38, 40, 41). Patients living alone are more likely to feel isolated and anxious, and this emotional burden may exacerbate their sleep disorders (39, 42–45). Therefore, living alone is not only a sociological factor but also reflects the vulnerability of patients’ quality of life and postoperative recovery.

Patients living in urban areas are exposed to a range of environmental stressors, including noise pollution, light pollution, and air pollution (46–48). These environmental factors can affect patients’ sleep quality in several ways, particularly during the postoperative recovery phase (49, 50). Higher noise levels and light pollution in urban areas may decrease sleep quality, disrupt biological clocks and sleep cycles, and increase the risk of PSD (51–54). Additionally, air pollution and the urban heat island effect may slow the recovery process and increase the incidence of postoperative complications (55, 56). Therefore, the living environment plays a significant moderating role in the development of PSD after TKA.

The findings of these social and environmental factors highlight that social support and environmental conditions are just as important as medical treatment during postoperative recovery. Therefore, these factors should be considered when developing postoperative interventions to ensure a more personalized care strategy.

### Validation of clinical factors and the benefits of machine learning models

Besides the two innovative factors—living alone and living in the city—our study also confirmed the role of traditional clinical factors in predicting postoperative sleep disorders. For example, the VAS score (postoperative pain score) is a significant risk factor for PSD. High VAS scores are associated with poorly managed postoperative pain, and persistent pain not only affects sleep quality but may also impact mood and recovery (57–60). Therefore, managing postoperative pain is crucial to reducing the risk of PSD.

Additionally, postoperative anxiety scores are identified as significant predictors. Postoperative anxiety exacerbates patients’ pain perception and affects their psychological state, thereby increasing the incidence of sleep disorders. Our study finds that anxiety symptoms are strongly associated with sleep disorders, indicating the need for effective management of anxiety symptoms in postoperative patients to reduce the risk of sleep disorders.

Age is another known influencing factor, as patients’ physiological conditions and rehabilitation capacity change with age. Older patients are at higher risk for comorbidities, such as hypertension and diabetes, which increase the incidence of postoperative sleep disorders (61–63). Our findings confirm the importance of age in postoperative sleep disorders and suggest that elderly patients require special attention for postoperative care and sleep health.

### Applications and benefits of machine learning models

Another innovation in this study is the use of ten machine learning models to analyze the data, including Logistic Regression, SVM, GBM, and Random Forest. Compared to traditional statistical methods, ML handles non-linear relationships and extracts key factors from complex multidimensional data. Through the comparative evaluation of these models, we identify the GBM as the best performer, with high accuracy and sensitivity.

Our research highlights the significant potential of ML in medical prediction, particularly for complex health issues like PSD. By integrating various ML algorithms, we can accurately identify high-risk patients for PSD and offer personalized clinical intervention recommendations. For instance, using our model, clinicians can identify high-risk patients early and implement appropriate management strategies, such as pain control, anxiety management, and adjustments to the living environment.

### Clinical application and deployment

The findings of this study have significant implications for clinical practice. The predictive model can be integrated into the preoperative assessment process for TKA patients. By using readily available clinical and social data, clinicians can identify patients at high risk for PSD prior to surgery, enabling proactive and personalized management strategies. For example, high-risk patients can be referred to prehabilitation programs focused on pain and anxiety management and offered counseling on sleep hygiene. Postoperatively, these patients can be monitored more closely, and non-pharmacological interventions (e.g., minimizing nighttime disruptions, cognitive behavioral therapy for insomnia) can be started early.

Importantly, our model identifies modifiable risk factors, such as postoperative VAS and anxiety, suggesting that PSD is a largely preventable complication. The model should not be viewed as a deterministic prognosis but as a tool for risk stratification that identifies specific areas for intervention. By effectively managing pain and addressing anxiety during the perioperative period, the incidence and severity of PSD can be significantly reduced. This model represents a shift from reactive treatment to proactive prevention, providing a pathway for improving postoperative care and outcomes.

Although the ML model shows promise, its successful deployment in clinical practice requires several key considerations. First, the model must be integrated into existing clinical workflows and decision-making systems to facilitate its use by healthcare professionals. Training and adaptation to various clinical settings are essential for effective use.

From a technical standpoint, the model should be scalable and capable of processing large volumes of patient data in real-time without excessive computational requirements. It is also crucial to validate the model across various hospitals and patient populations to ensure its generalizability and applicability.

While this study offers significant innovative value, several limitations should be considered to contextualize the findings and guide future research. First, the single-center, retrospective design, while providing a robust initial dataset, may limit the generalizability of our model to other healthcare settings and patient populations. This design also carries an inherent risk of unmeasured confounders. Therefore, external validation in multi-center, prospective cohorts is a necessary next step. Second, the predictive scope of our model is limited by the variables available in our dataset. While we include a range of clinical and socio-environmental factors, other potentially influential variables, such as genetic predispositions, detailed psychosocial characteristics, and environmental factors, are not accounted for. Furthermore, the lack of long-term follow-up data beyond 1 month limits our understanding of the model’s ability to predict persistent sleep disturbances. Future studies incorporating these omitted factors and longer-term outcomes are crucial for enhancing the model’s comprehensiveness and clinical relevance. Finally, in terms of model evaluation, our analysis primarily focuses on discriminative performance (the ability to distinguish between PSD and non-PSD patients). We do not formally assess model calibration, which measures the accuracy of predicted risk probabilities. As calibration is a key metric for evaluating the clinical usefulness of a predictive model, investigating it remains an important area for future work. Additionally, despite our efforts to conduct subgroup analyses, potential biases arising from imbalances in sociodemographic factors may persist, affecting model performance. Further validation in larger and more diverse populations is recommended to ensure fairness and generalizability.

## Conclusion

This study developed a ML-based model for predicting PSD in patients following TKA. By analyzing factors such as age, smoking history, VAS score, and anxiety score, we identified key predictors of PSD. The GBM model showed the best predictive efficacy, with high accuracy and sensitivity. We further enhanced the model’s interpretability using SHAP methodology, enabling clinicians to visualize the specific contribution of each factor to the prediction, facilitating preoperative risk stratification and personalized interventions.

Additionally, our study identified two socio-environmental factors—living alone and living in the city—that have not been sufficiently explored in the literature. Patients living alone face greater postoperative challenges due to lack of family support, while those living in urban areas are more exposed to environmental stressors, such as noise and light pollution, which exacerbate the risk of PSD. These findings offer new insights for clinical interventions, emphasizing the importance of social and environmental factors in postoperative care.

Future studies should validate this model across diverse populations, expand its applicability, and incorporate additional factors such as genetic background and long-term follow-up data to enhance its predictive ability and clinical value.

## Data Availability

The original contributions presented in this study are included in this article/Supplementary material, further inquiries can be directed to the corresponding authors.
