# Supplementary material for: A machine learning approach to predict postoperative sleep disturbance after total knee arthroplasty: a comparative study of multiple algorithms
Source: Front Med (Lausanne). 2025 Nov 5;12:1699842. doi: 10.3389/fmed.2025.1699842 (PMC12627052; doi:10.3389/fmed.2025.1699842)
Supplement: Supplementary file 1 [file Table_1.docx]

Supplementary Table1 Summary of Missing Data in model development dataset

| Variables | Number | Missing data | Missing percent |
| --- | --- | --- | --- |
| Cr (mg/dL) | 500 | 5 | 0.99 |
| D_dimer (mg/L) | 502 | 3 | 0.59 |
| CRP (mg/L) | 502 | 3 | 0.59 |
| TB (mmol/L) | 502 | 3 | 0.59 |
| APTT (s) | 502 | 3 | 0.59 |
| PT (s) | 502 | 3 | 0.59 |
| Preoperative Anxiety | 492 | 13 | 2.57 |
| Preoperative Depression | 492 | 13 | 2.57 |
| Preoperative Womac | 477 | 28 | 5.54 |
| VAS one month postoperative | 484 | 21 | 4.16 |
| Womac one month postoperative | 461 | 44 | 8.71 |
| Anxiety one month postoperative | 458 | 47 | 9.31 |
| Depression one month postoperative | 458 | 47 | 9.31 |
| One month postoperative Knee Range of Motion | 477 | 28 | 5.54 |
